# Association between serum uric acid and cardiovascular risk in nonhypertensive and nondiabetic individuals: The Taiwan I-Lan Longitudinal Aging Study

**DOI:** 10.1038/s41598-018-22997-0

**Published:** 2018-03-27

**Authors:** Chun-Chin Chang, Cheng-Hsueh Wu, Li-Kuo Liu, Ruey-Hsing Chou, Chin-Sung Kuo, Po-Hsun Huang, Liang-Kung Chen, Shing-Jong Lin

**Affiliations:** 1 Division of Cardiology, Taipei, Taiwan; 2 Division of Endocrinology and Metabolism, Department of Medicine, Taipei, Taiwan; 3 Department of Critical Care Medicine, Taipei, Taiwan; 4 Center for Geriatrics and Gerontology, Taipei, Taiwan; 5 Healthcare and Services Center, Taipei Veterans General Hospital, Taipei, Taiwan; 6 Cardiovascular Research Center, Taipei, Taiwan; 7 Institute of Clinical Medicine, Taipei, Taiwan; 8 Aging and Health Research Center, Taipei, Taiwan; 9 Institute of Public Health, National Yang-Ming University, Taipei, Taiwan; 10 Taipei Medical University, Taipei, Taiwan

## Abstract

Serum uric acid level is a risk factor for cardiovascular disease (CVD). However, whether it is an independent risk factor or not remains controversial. We analyzed the association between serum uric acid level and cardiovascular risk. In total, 973 nonhypertensive and nondiabetic participants in the I-Lan Longitudinal Aging Study were eligible for this study. Subjects were divided into tertiles according to uric acid levels. The 10-year cardiovascular risk was calculated using Framingham risk score (FRS). Study subjects in the highest tertile of serum uric acid level were older, more likely to be male, and had higher systolic blood pressure, body mass index, carotid artery intima–media thickness and serum triglyceride, high-sensitivity C-reactive protein, and low-density lipoprotein cholesterol levels and lower serum high-density lipoprotein cholesterol levels (all p < 0.05). Subjects in the highest tertile had significantly higher FRS (p < 0.001). After adjusting for other risk factors, serum uric acid level remained associated significantly with the FRS (p < 0.05). In binary logistic regression analysis, the serum uric acid level was an independent predictive factor for high (≥20%) FRS (odds ratio 1.33, 95% confidence interval 1.10–1.68). These findings warrant attention to this cardiovascular risk factor in apparently healthy adults.

## Introduction

An increased serum uric acid level is a common finding in patients with hypertension, insulin resistance, obesity, and cardiovascular disease (CVD). Moreover, high uric acid levels have been found to be associated with higher incidences of diabetes, hypertension, and chronic kidney disease. An elevated serum uric acid level has been considered to be a risk factor for CVD. However, debate remains about whether such elevation is an independent predictor of cardiovascular risk. Uric acid elevation has been proposed to be a compensatory mechanism to counteract oxidative damage related to atherosclerosis^1^. Nevertheless, the hypothesis that reduction of the serum uric acid level could prevent CVD has not been tested. The serum uric acid level probably reflects and integrates different risk factors and their possible interactions.

In the current study, we investigated the association between serum uric acid concentration and cardiovascular risk in nonhypertensive and nondiabetic individuals, with the aim of minimizing potential unwanted interactions. An understanding of the clinical significance of this relationship may prompt earlier surveillance of the serum uric acid level in asymptomatic individuals, and result in the identification of populations at high risk of CVD.

## Results

Data from 973 nonhypertensive and nondiabetic ILAS participants (46.8% men, mean age 61.7 ± 8.6 years, range 50–89 years) were analyzed. Table 1 summarizes the demographic and clinical characteristics of the study subjects. The subjects were divided into tertiles according to serum uric acid concentration (first tertile, <5.0 mg/dl; second tertile, 5.0–6.1 mg/dl; third tertile, >6.1 mg/dl).

**Table 1:** Characteristics of study participants according to uric acid tertile (mg/dl).

	1st tertile < 5.0	2nd tertile 5.0–6.1	3rd tertile > 6.1	*p* value
	(*n* = 313)	(*n* = 307)	(*n* = 353)	
Framingham CVD risk score (%)	8.8 ± 8.5	14.2 ± 11.7	20.4 ± 14.4	<0.001
***Framingham CVD risk score components***				
Age (years)	60 ± 8.0	62 ± 8.0	63 ± 9.0	0.005
Male, *n* (%)	55 (17.6)	136 (44.3)	264 (74.8)	<0.001
HDL-C (mg/dl)	62 ± 14	56 ± 13	52 ± 13	<0.001
Total cholesterol (mg/dl)	200 ± 34	201 ± 34	202 ± 36	0.878
SBP (mmHg)	122 ± 15	125 ± 16	128 ± 16	0.002
Smoking	38 (12.1)	100 (32.4)	171 (48.4)	<0.001
***Covariates***				
BMI (kg/m^2^)	23 ± 3.0	24 ± 3.2	25.0 ± 3.0	<0.001
LDL-C (mg/dl)	118 ± 31	125 ± 30	126 ± 33	<0.001
Triglycerides (mg/dl)	96 ± 51	110 ± 57	139 ± 89	<0.001
HbA1c (%)	5.8 ± 0.7	5.8 ± 0.6	5.7 ± 0.5	0.189
cIMT (mm)	0.63 ± 0.11	0.67 ± 0.14	0.68 ± 0.13	<0.001
hsCRP (mg/dl)	0.16 ± 0.32	0.21 ± 0.56	0.21 ± 0.36	0.004
Uric acid (mg/dl)	4.2 ± 0.6	5.5 ± 0.3	7.2 ± 1.0	<0.001

Values are mean ± standard deviation or *n* (%). BMI = body mass index, cIMT = carotid intima–media thickness, CVD = cardiovascular disease, HbA1c = glycated hemoglobin, HDL-C = high-density lipoprotein cholesterol, hsCRP = high-sensitivity C-reactive protein, LDL-C = low-density lipoprotein cholesterol, SBP = systolic blood pressure.

Study participants in the third tertile of serum uric acid level were older, more likely to be male, and had higher SBP, BMI, and serum triglyceride and LDL-C levels and lower HDL-C levels (all *p < *0.05). Subjects with higher serum uric acid levels also had significantly enhanced high-sensitivity C-reactive protein levels and increased cIMT (both *p* < 0.005).

Table 2 shows coefficients of correlation between the serum uric acid level and the FRS and other cardiovascular risk factors. The serum uric acid level was correlated positively with the FRS, age, SBP, BMI, triglyceride level, LDL-C level, high-sensitivity C-reactive protein level, smoking, and cIMT (all *p* < 0.01), but negatively with the HDL-C level and glomerular filtration rate (GFR) (both *p* < 0.01). In multivariate analysis, the correlation between the serum uric acid level and the FRS remained significant after adjustment for age, BMI, SBP, triglyceride level, LDL-C level, HDL-C level, GFR, high-sensitivity C-reactive protein level, cIMT, and smoking (*p = *0.002; Table 3, model 3).

**Table 2:** Coefficients of correlation between the serum uric acid level and cardiovascular risk variables.

	FRS	Age	HDL	TC	SBP	BMI	TG	LDL	GFR	hsCRP	Smoking	cIMT
Uric acid	0.466*	0.102*	−0.321*	−0.005	0.124*	0.274*	0.277*	0.112*	−0.339*	0.132*	0.240*	0.164*
FRS	1	0.524*	−0.382*	0.008	0.482*	0.210*	0.265*	0.163*	−0.553*	0.109*	0.469*	0.348*

BMI = body mass index, cIMT = carotid intima–media thickness, FRS = Framingham risk score, GFR = glomerular filtration rate, hsCRP = high-sensitivity C-reactive protein, HDL = high-density lipoprotein, LDL = low-density lipoprotein, SBP = systolic blood pressure, TC = total cholesterol, TG = triglycerides. *p < 0.01 (two-tailed).

**Table 3:** Associations of the Framingham risk score with the uric acid level in multivariate analyses.

Dependent variable FRS	Standardized coefficient β	*p* value
Model 1	0.232	<0.001
Model 2	0.231	<0.001
Model 3	0.057	0.002

Model 1: adjusted for age, HDL, and SBP.

Model 2: adjusted for age, HDL, SBP, BMI, and TG.

Model 3: adjusted for age, HDL, SBP, BMI, TG, GFR, hsCRP, and smoking.

BMI = body mass index, FRS = Framingham risk score, GFR = glomerular filtration rate, hsCRP = high-sensitivity

C-reactive protein, HDL = high-density lipoprotein, SBP = systolic blood pressure, TG = triglycerides.

The study subjects were separated into three groups according to the FRS. Two hundred forty-three subjects were assigned to the high-risk group (FRS ≥ 20%), 256 were assigned to the intermediate-risk group (FRS 10–20%), and 474 were assigned to the low-risk group (FRS ≤ 10%). The serum uric acid level differed significantly among groups (*p* < 0.001); it was significantly higher in the high-risk group than in the intermediate-risk and low-risk groups [6.5 mg/dl vs. 6.1 mg/dl (*p* = 0.001) and 5.1 mg/dl (*p* < 0.001), respectively].

Binary logistic regression analysis was also performed to define the predictive value of the serum uric acid level for high (≥20%) FRS. Age, BMI, SBP, HDL-C level, triglyceride level, and GFR were included in the analysis. A high serum uric acid level was found to be associated independently with a high FRS (odds ratio 1.33, 95% confidence interval 1.10–1.68; Table 4).

**Table 4:** Risk factors associated with high Framingham risk scores in binary logistic regression analysis.

	Univariate	*p* value	Multivariate	*p* value
	odds ratio (95% CI)		odds ratio (95% CI)	
Age, 1SD = 8.6 years	2.87 (2.43–3.40)	<0.001**	1.94 (1.50–2.50)	<0.001**
HDL-C, 1SD = 14 mg/dl	0.42 (0.35–0.51)	<0.001**	0.38 (0.28–0.50)	<0.001**
SBP, 1SD = 16 mmHg	2.13 (1.82–2.50)	<0.001**	2.75 (2.14–3.54)	<0.001**
BMI, 1SD = 3.3 g/cm^2^	1.28 (1.11–1.47)	0.001*	1.73 (1.29–2.32)	<0.001**
TG, 1SD = 73 mg/dl	1.50 (1.30–1.74)	<0.001**	1.11 (0.91–1.35)	0.279
GFR, 1SD = 27 ml/min	0.25 (0.20–0.32)	<0.001**	0.24 (0.16–0.35)	<0.001**
hsCRP, 1SD = 0.45 mg/dl	1.12 (0.97–1.29)	0.114		
Uric acid, 1SD = 1.4 mg/dl	2.16 (1.84–2.52)	<0.001**	1.33 (1.10–1.68)	<0.017*

BMI = body mass index, CI = confidence interval, GFR = glomerular filtration rate, hsCRP = high-sensitivity C-reactive protein, HDL-C = high-density lipoprotein cholesterol, SBP = systolic blood pressure, TG = triglycerides.

*p < 0.05, **p < 0.001.

## Discussion

The main question addressed in this study was whether the serum uric acid level is an independent risk factor for CVD in nonhypertensive and nondiabetic individuals. Our findings demonstrate that elevated serum uric acid levels were associated independently with high FRS after adjustment for other potential confounding risk factors. Of note, the uric acid level was also associated with the high-sensitivity C-reactive protein level and cIMT, markers of systemic inflammation and preclinical atherosclerosis. These findings provide pivotal evidence that elevated uric acid concentrations may increase CVD risk and represent a potential therapeutic target in the primary prevention of CVD.

Uric acid is a major product of purine metabolism. In adults, the serum uric acid level varies with body weight, height, blood pressure, renal function, and alcohol intake^2^. Uric acid levels are also affected by numerous conditions, including obesity, hypertension, diabetes, hyperlipidemia, and renal disease, and by diuretic use^3,4^. Moreover, a previous report showed that hyperuricemia was associated with insulin resistance and metabolic syndrome in essentially health subjects^5^.

The association between uric acid and CVD has been discussed since the 1950s^6^. However, the clinical relevance of uric acid in CVD remains controversial. The National Health and Nutrition Examination Survey I Follow-Up Study, in which 5,926 subjects with 16.4 years of follow up were enrolled, demonstrated that increased serum uric acid levels were associated significantly with a higher risk of cardiovascular mortality^7^. After controlling for potential risk factors, this association persisted, even in subjects with low cardiovascular risk. Similar results were obtained in a prospective study of middle-aged Finnish men^8^. The uric acid concentration is a strong predictor of cardiovascular mortality in men without clinical CVD and diabetes. Data from another epidemiological longitudinal survey also showed serum uric acid is a predictor of long-term incidence of CVD events, deaths and all-cause mortality^9^. In addition, a gender difference in the association between serum uric acid and CVD incidence has been reported. The association was more strongly in women than in men^10^. Our study extends previous findings to further support the link between a high serum uric acid level and CVD.

In contrast, some epidemiological studies have indicated that the serum uric acid level is not independent of other established risk factors for CVD^11–13^ In a community-based, prospective observational study in which 6,763 Framingham Heart Study participants were followed, the positive association between the serum uric acid level and cardiovascular events was not sustained after accounting for other risk factors^11^. The greatest difficulty in clarifying this debate stems from the frequent association of the circulating uric acid concentration with other cardiovascular risk factors, such as hypertension, chronic kidney disease, metabolic syndrome, diabetes, and smoking. Accordingly, previous studies have been limited in their ability to elucidate the causal role of uric acid in CVD.

Several experimental and clinical studies have refueled the debate. Uric acid has been found to impair nitric oxide synthesis, resulting in vascular endothelial dysfunction^14^. Not surprisingly, similar effects were observed in patients with hyperuricemia. Flow-mediated vasodilation, representative of endothelial function, was significantly lower in hyperuricemic patients than in a control group^15^. Endothelial dysfunction, characterized by the alteration of the vascular lining, is prone to pro-thrombotic, pro-inflammatory, and pro-constrictive. Our recent data suggest an association between a previous history of gout attack and the risk of deep vein thrombosis^16^. Furthermore, endothelial dysfunction is associated with arterial stiffness and the development of hypertension. Emerging evidence also demonstrates links between the uric acid level and wave reflection and arterial stiffness^17,18^.

The findings of numerous studies support the relationship between serum uric acid and CVD. Zoppini *et al*.^19^ reported that higher uric acid levels were associated with an increased risk of cardiovascular mortality in patients with type 2 diabetes after adjustment for other risk factors, including age, sex, BMI, smoking, hypertension, dyslipidemia, diabetes duration, HbA1c level, medication use, and renal function. In this large cohort study, we demonstrated that enhanced uric acid levels were associated with increased systemic inflammation, preclinical atherosclerosis, and future cardiovascular risk. However, whether reduction of the uric acid concentration in hyperuricemic patients could have beneficial effects, reducing cardiovascular morbidity and mortality, remains unknown. Further large prospective studies are warranted to verify this hypothesis.

Some possible limitations of this study should be mentioned. First, given the cross-sectional design, we could not establish a causal relationship between uric acid elevation and CVD. The study results obtained from an observational analysis. Moreover, our participants were healthier than the general population of community-dwelling older adults because we excluded those with hypertension, diabetes, and disability. Finally, a future prospective study is needed to determine whether the serum uric acid level is an independent predictor of long-term cardiovascular outcomes, and whether its reduction could reduce the occurrence of adverse cardiovascular events.

In conclusion, the serum uric acid level was associated significantly with the FRS in nonhypertensive and nondiabetic individuals. Further prospective studies are required to determine whether reduction of the uric acid concentration has beneficial effects, reducing cardiovascular risk.

## Methods

### Study design and population

The I-Lan Longitudinal Aging Study (ILAS) is a community-based aging cohort study conducted in I-Lan County, Taiwan^20^. Community-dwelling adults aged > 50 years were randomly sampled through the household registries of the county government in Yuanshan Township. Selected residents were invited by the research team to participate, and informed consent was obtained from all participants. The inclusion criteria were: (i) residence in I-Lan County with no plan to move in the near future and (ii) age >50 years. Respondents that met any one of the following conditions were excluded from the study: (i) inability to communicate with the interviewer; (ii) poor functional status that could lead to failure of evaluation, such as inability to complete a 6-m timed walk within a reasonable period of time; (iii) limited life expectancy (<6 months) because of major illness; (iv) presence of an implant contraindicating magnetic resonance imaging; and (v) current institutionalization.

In total, 1,798 inhabitants of I-Lan County were enrolled. After the further exclusion of participants with hypertension and/or diabetes (214 had hypertension and diabetes, 526 had hypertension, and 85 had diabetes), 973 participants remained eligible for this study. The institutional review board of National Yang-Ming University approved the research as a whole. All research was performed in accordance with relevant regulations.

### Demographic data collection and physical examination

Participants’ medical histories, including information about conventional cardiovascular risk factors, and current drug treatments were obtained during personal interviews and from medical files. Each participant’s weight, height, and waist circumference were measured, and the body mass index (BMI) was calculated. Brachial blood pressure was accessed with a mercury sphygmomanometer after patients sat for ≥15 min. The average of three systolic blood pressure (SBP) measurements was used for the analysis.

### Framingham risk score

The Framingham risk score (FRS) is a widely accepted formulation for CVD prediction that has been validated in large population studies^21^. The Framingham Heart Study group provided several risk prediction equations for CVD, coronary heart disease, congestive heart failure, and atrial fibrillation. Among these equations, we adapted the equation used to calculate the 10-year risk of CVD development for this study. The suggested equation considered age, sex, high blood pressure, smoking, hyperlipidemia, and diabetes to be the major risk factors for CVD development. The equation incorporates multivariate analysis with calculation of a regression coefficient for each possible risk factor.

### Carotid ultrasonography

Carotid ultrasonography was performed with an ultrasound system (GE LOGIQ 400 PRO; GE, Cleveland, OH, USA). A well-trained technician performed measurements for all participants. Images were obtained bilaterally of the proximal to distal common carotid artery (CCA), the carotid bifurcations, and the origin of the internal carotid arteries. The carotid intima–media thickness (cIMT) was measured at a site with no discrete plaque along a 10-mm-long segment of the far wall of the CCA. The average of the bilateral cIMTs was calculated for analysis^22^.

### Laboratory measurement

All blood samples were drawn with the participant in the seated position after a 10-h overnight fast. Serum concentrations of glucose, uric acid, total cholesterol, triglycerides, low-density lipoprotein cholesterol (LDL-C), high-density lipoprotein cholesterol (HDL-C) and high-sensitivity C-reactive protein were determined using an automatic analyzer (ADVIA 1800; Siemens, Malvern, PA, USA). Whole-blood glycated hemoglobin A1c (HbA1c) was measured by an enzymatic method using the Tosoh G8 HPLC Analyzer (Tosoh Bioscience, Inc., San Francisco, CA, USA).

### Statistical analysis

The complete dataset was analyzed, and results were expressed as means ± standard deviations or as frequencies and percentages. Comparisons among the three groups were made by analysis of variance, the Kruskal–Wallis test, or the chi-squared test, as appropriate. The chi-squared test was used for subgroup comparisons of categorical variables. Linear regression analysis was performed to evaluate the association between the serum uric acid level and FRS. Data were analyzed using SPSS software (version 20; SPSS Inc., Chicago, IL, USA). Two-tailed *p* values < 0.05 were considered to indicate statistical significance.
